# Expanding Praziquantel (PZQ) Access beyond Mass Drug Administration Programs: Paving a Way Forward for a Pediatric PZQ Formulation for Schistosomiasis

**DOI:** 10.1371/journal.pntd.0004946

**Published:** 2016-09-22

**Authors:** Amaya L. Bustinduy, Jennifer F. Friedman, Eyrun Floerecke Kjetland, Amara E. Ezeamama, Narcis B. Kabatereine, J. Russell Stothard, Charles H. King

**Affiliations:** 1 Department of Clinical Research, London School of Hygiene and Tropical Medicine, London, United Kingdom; 2 Center for International Health Research, Rhode Island Hospital and Alpert Medical School of Brown University, Providence, Rhode Island, United States of America; 3 Norwegian Centre for Imported and Tropical Diseases, Department of Infectious Diseases Ullevaal, Oslo University Hospital, Oslo, Norway; 4 Discipline of Public Health Medicine, Nelson R. Mandela School of Medicine, College of Health Sciences, University of KwaZulu-Natal, Durban, South Africa; 5 Department of Epidemiology and Biostatistics University of Georgia, Athens, Georgia, United States of America; 6 Schistosomiasis Control Initiative, Imperial College London, London, United Kingdom; 7 Department of Parasitology, Liverpool School of Tropical Medicine, Liverpool, United Kingdom; 8 Center for Global Health and Diseases, Case Western Reserve University, Cleveland, Ohio, United States of America; Institute of Cell Biology, ITALY

## Overview

Treating preschool age children (PSAC) with schistosomiasis remains a challenge. Without a pediatric praziquantel (PZQ) formulation, the inclusion of this age group in control programs is limited, and general access to treatment in routine care settings is severely bottlenecked. There are, however, current platforms that target PSAC in primary health care such as the integrated management of childhood illnesses (IMCI), which could integrate PZQ in their portfolio and deliver a pediatric PZQ formulation when available. In addition, other age groups such as school-aged children (SAC) could also benefit from the IMCI’s successful strategy and be treated in health centers using a similar approach. This Viewpoint article reports a summary of a symposium held at the American Society of Tropical Medicine and Hygiene national meeting in 2014 that brought together six experts in different areas in the field of pediatric schistosomiasis to form a working group that could provide recommendations for the inclusion of PSAC in the IMCI and other existing preschool outreach programs. This was to develop and adapt methodologies to fill existing gaps left by current mass drug administration (MDA) programs and synergize efforts for schistosomiasis control more broadly. Foremost, this includes a better definition of subclinical disease in young children to integrate into ICMI guidelines and further demonstration of the benefit of expanded access of treatment to children of all ages by encouraging universal access.

## The Double Treatment Gap for Schistosomiasis

Over 123 million children suffer from schistosomiasis worldwide, but only SAC are presently targeted for MDA with PZQ treatment in WHO-led preventive chemotherapy (PC) efforts [1]. This is despite growing evidence that in some endemic foci a majority of PSAC become infected and can develop early disease [2,3]. The need to include the vulnerable PSAC group in MDA in order to prevent and avert early-onset morbidity is acknowledged by WHO, and work is in progress to develop a workable pediatric PZQ formulation [4]. Nevertheless, ongoing MDA programs only cover 34.6% of SAC with standard tablets (600 mg), meaning at least 79 million SAC go untreated [5], contributing to the existing double PZQ treatment gap (untreated PSAC and SAC). There is commitment to scale up this figure in the next few years, as PZQ donation is soon to reach more than 100 million SAC per year [6].

The IMCI is one of WHO/UNICEF’s most successful strategies in reducing mortality in children under five years of age [7]. Since it was first conceived in 1997, it has provided over 75 countries with easy-to-use tools for primary care health workers to promptly identify and treat life-threatening conditions such as pneumonia, diarrhea, malaria, measles, and malnutrition. The IMCI has quickly expanded to now include health prevention measures such as Vitamin A supplementation, vaccination, and treatment for soil-transmitted helminths (STHs) in endemic areas. Its success relies in part on the rapid and effective syndromic approach to treatment using standard case management, permitting local adaptation in accordance with regional variations in the prevalence of deadly diseases. As the IMCI expands to include other health threats to children under five, pediatric schistosomiasis, a devastating, disabling neglected tropical disease (NTD), is the next candidate that could benefit from inclusion in its successful strategy.

**Fig 1:**
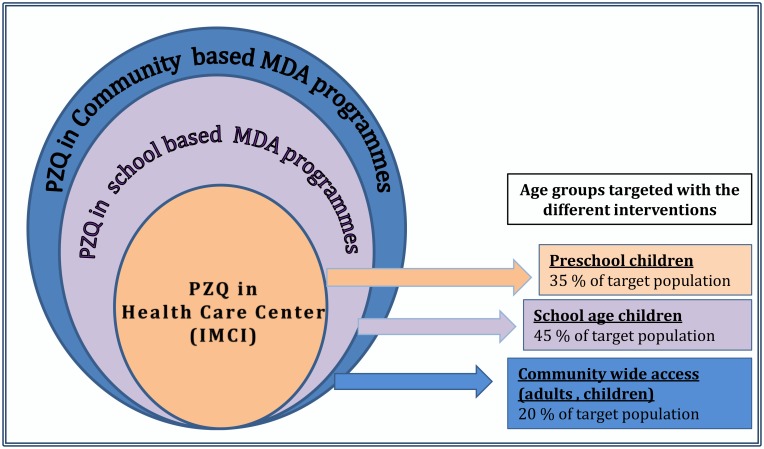
Proposed PZQ delivery strategies at different levels of care and the targeted age groups for each one.

In most endemic areas, individual case management for clinical schistosomiasis is not happening, because the disease is poorly recognized in younger children [8]. This is often due to the first-level health worker’s lack of understanding of the range of schistosomiasis clinical manifestations. Furthermore, there is a widely accepted perception that disease will ultimately be “treated” through MDA programs during school age years. This resulting double treatment gap (PSAC and SAC) could be addressed with the integration of public health approaches (e.g., MDA) with individual case management interventions. This would increase the delivery of PZQ at the primary care level, as occurred in the case of mebendazole therapy for STHs [9]. Integrated early treatment could have programmatic advantages for health ministries and, more importantly, could also prevent schistosomiasis-associated morbidity and downstream complications later in life (Fig 1).

## Preventable Morbidities with Earlier PZQ Treatment

### Anemia, growth, and nutrition

Nutritional status is considered a key “vital sign” in the assessment of the pediatric patient, integrating both long- and short-term insults to health. Schistosomiasis is well known to cause deficits in linear growth, leading to chronic undernutrition (stunting) as well as acute undernutrition (wasting) [1]. As practitioners gain expertise in identifying children who have experienced any kind of growth insult, there is an opportunity through the IMCI global strategy to include PZQ as a key intervention to treat one of the many causes of undernutrition among children living in poverty [10]. Use of this drug has the greatest nutritional benefit for children who are undernourished at the time of treatment [11]. Also of note, studies have demonstrated that children experiencing the greatest morbidity due to schistosomiasis have elevated levels of pro-inflammatory cytokines that lead to anorexia and cachexia, which may limit the benefits of any food supplement interventions provided for undernutrition in this setting [12].

Provision of PZQ as part of IMCI would also provide an opportunity to treat well-documented, schistosomiasis-associated anemia [1]. Improvements in hemoglobin with the treatment of schistosomiasis likely occurs through decreasing occult blood loss, which occurs at higher intensities of infection, and amelioration of anemia of inflammation [13]. The role of anemia of inflammation in schistosomiasis is relevant, as iron absorption from the gut is decreased and iron is shunted from bio-available forms to storage forms. The provision of iron in this context, therefore, will have a dampened effect. Thus, treatment of schistosomiasis will have dual benefit by treating the underlying inflammation due to infection and maximizing children’s ability to benefit from iron supplementation.

### Female and male urogenital schistosomiasis

Females with schistosomiasis carry an additional burden of reproductive tract disease that has been neglected in clinical management since its discovery more than 120 years ago. Female Genital Schistosomiasis (FGS) causes bleeding and three distinct lesions: grainy, sandy patches, rubbery papules, and homogenous yellow patches. Studies in Tanzania and Zimbabwe found that women with FGS have a 3- to 4-fold increased risk of having HIV; they also seem to have increased secondary infertility, contact bleeding, pain, and stress incontinence [14]. Girls as young as 10–12 years old have been found to have malodorous discharge, itching, and bloody discharge (even before menstrual periods) [15]. Furthermore, gynecological lesions were found to be irreversible in adult women even after several rounds of treatment with PZQ. Males are also affected and could be important for male–female HIV transmission [16].

The perfect timing of treatment for the prevention of lesions (and HIV) should therefore also be explored, but it is within reason to assume that preschool treatment could have a tangible impact on preventing genital lesions [15].

### Cognition

The evidence for educational or cognitive benefit of deworming of any kind is currently debated [2,7]. The expectation of educational and cognitive benefit is implied by mass deworming programs, making future randomized controlled trials to provide conclusive evidence ethically nonpermissive. Ongoing meta-analysis work is pooling data across 30 individual studies to evaluate the cognitive impact of schistosomiasis in SAC (Ezeamama et al., submitted for publication). Outcomes evaluated include educational loss in two domains (attendance and achievement) and cognitive performance in four domains (learning, memory, attention, and intelligence). This effort suggests that schistosomiasis infection is associated with significant educational loss and significant deficits in learning and memory, but not innate intelligence or attention. Therefore, early treatment of children in *Schistosoma*-endemic regions may be beneficial for preventing downstream educational loss and cognitive impairment.

### Early advanced disease

Epidemiological studies with global positioning system data loggers have revealed that PSAC can have surprisingly high levels of daily and cumulative water contact within the first three years of life [17]. With more sensitive diagnostics, such as serology, these early-stage infections can be better revealed, thereby providing better causal associations with direct markers of disease, e.g., fecal occult blood or microhematuria [1], along with more subtle aspects of morbidity, e.g., anemia and organomegaly, in case-control studies. Recent reports using ultrasonography highlight that advanced disease such as chronic fibrosis can be traced back to preschool childhood, which further evidences why earlier access to PZQ is vital [18].

## Pediatric PZQ Formulation: When Will It Become Available?

The safety of PZQ in PSAC has been widely established across *Schistosoma* species [3,19,20], but recent PZQ pharmacokinetic and pharmacodynamic data suggest that current recommended dosing is too low at 40 mg/kg and that higher dosing is needed for young children [21]. However, there are issues regarding the operational difficulties and choking risks of crushing PZQ tablets across both clinical practice as well as MDA programs. The development of the very needed pediatric PZQ formulation by Merck-Serono is currently undergoing human trials after successfully completing the preclinical developmental stage [22]. Their target is to register and launch a new pediatric formulation in the first endemic countries by 2019. Until then, treating PSAC will rely solely on PZQ crushed tablets, and currently WHO is not formally capturing this aspect of treatment within the PC database [4].

## Conclusions

We have identified important treatment gaps in the ongoing MDA strategy for schistosomiasis that leave infected children untreated. A synergistic approach to schistosomiasis control could integrate PZQ delivery through ongoing primary health care strategies (IMCI) both for PSAC as well as SAC, with ongoing MDA efforts that currently exclude PSAC. When the PZQ pediatric formulation becomes available, a solid clinical care pathway with an optimized dosing strategy needs to be in place to allow an efficient delivery into existing programs.

**Fig 2:**
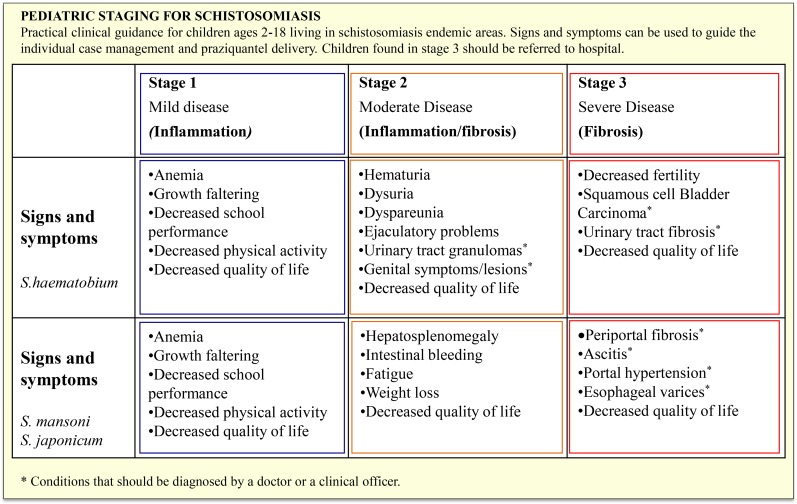
Pediatric clinical staging for the different clinical manifestations of schistosomiasis in childhood.

We propose a step-wise approach to identify and treat children of any age with schistosomiasis through (1) *Identification of schistosomiasis individual cases* using a detailed clinical classification that could be taught to health care workers so they could identify children with suspected disease, and in areas where prevalence is known to be high, provision of treatment could be the first standard of care (Fig 2). This is important particularly for the nonspecific manifestations, such as anemia and growth retardation, that are highly prevalent in resource-limited environments where schistosomiasis coexists with other endemic diseases. In addition, the use of point-of-care (POC) *Schistosoma* diagnostics could become more widely available, allowing individualized test-and-treat approaches [23]. (2) *Access to PZQ treatment in the health center*. Through the national delivery of PZQ to health centers, trained staff can treat suspected schistosomiasis cases based on the clinical staging proposed. This applies to standard tablet formulation of PZQ, now widely available at low cost over the counter in pharmacies in several countries. Moreover, the surplus from MDA programs could be given to health centers to allow year-round continuity of treatment. (3) *Integration of PZQ within the IMCI when the PZQ pediatric formulation becomes available*. PC to PSAC can be synergized with other preventive measures (e.g., vaccines, vitamin A supplementation, deworming with mebendazole, and parental education to prevent re-infection) when a pediatric formulation is available. Meanwhile, while waiting for a pediatric formulation, the use of crushed tablets should be continued on a case-by-case basis, which is common practice when very young children need other essential drugs [24] and which can be done with adequate training and supervision by health workers. Antischistosomal treatment in PSAC should not be delayed. This simple change in tactics for schistosomiasis control will make use of all available tools, whether in the community (via IMCI) or by WHO-led efforts for PC through PZQ MDA programs: a strategy not only ethically correct, but also cost effective [25]. It is time to discard the common sit-and-wait approach for PSAC schistosomiasis and move toward an effective test-and-treat approach for PSAC.
